# Comparison of hydroxyapatite and fluoride oral care gels for remineralization of initial caries: a pH-cycling study

**DOI:** 10.1038/s41405-020-0037-5

**Published:** 2020-07-22

**Authors:** Bennett T. Amaechi, Parveez Ahamed AbdulAzees, Linda O. Okoye, Frederic Meyer, Joachim Enax

**Affiliations:** 1 Department of Comprehensive Dentistry, School of Dentistry, University of Texas Health San Antonio, 7703 Floyd Curl Drive, San Antonio, TX 78229-3900 USA; 2 Department of Restorative Dentistry, Faculty of Dentistry, College of Medicine, University of Nigeria, Enugu, Nigeria; 3 Dr. Kurt Wolff GmbH & Co. KG, Research Department, Johanneswerkstr. 34-36, 33611 Bielefeld, Germany

**Keywords:** Minimal intervention dentistry, Oral hygiene

## Abstract

**Objective:**

The present in vitro study investigated if simulated daily use of hydroxyapatite-based gel (15% HAP) remineralizes early caries lesions as effective as weekly use of high fluoride (12,500 ppm) concentration gel, comparing them with artificial saliva alone.

**Materials and methods:**

Three tooth blocks were produced from each of 20 bovine teeth. Caries-like lesion was created on each block by 4-day demineralization in acidified gel. The blocks were randomized into three remineralization groups (20 blocks/group); Hydroxyapatite-based gel (Karex gelée, 15% HAP, fluoride-free), fluoride-based gel (Elmex gelée, 12,500 ppm F^−^), and artificial saliva (AS). Remineralization was conducted using pH-cycling model for 28 days with storage in AS. The pH cycling model consisted of 2 h demineralization once daily for all groups, and 3 min HAP gel application once daily, 3 min fluoride gel application once weekly, or remain in AS only respectively. Baseline and post-test mineral loss were quantified using microradiography.

**Results:**

Paired t-tests (baseline vs. post-Test) indicated significant (*p* < 0.0001) remineralization in all groups. When compared against each other using Games-Howell’s multiple comparison test, no significant difference in remineralization was observed between the two gels, but both gels exhibited significantly (*p* < 0.001) higher percentage mineral gain (HAP:39 ± 7%; fluoride:41 ± 11%) than AS alone (6 ± 2%).

**Conclusion:**

Hydroxyapatite-based gel (15% HAP) was as effective as fluoride-based gel (12,500 ppm F^−^) in remineralizing initial caries lesion.

## Introduction

Dental caries is an oral disease that affects millions of individuals, both children and adults, worldwide.^1^ The latest data from 2015 report a caries incidence of 616 million on deciduous and permanent teeth worldwide.^1^ For many years, fluoride treatments such as varnishes, lozenges, and sealants have been used for caries prevention. However, it is now known that fluoride alone or in combination with chlorhexidine is not sufficient to prevent tooth decay, especially in high caries risk patients.^2,3^ Hausen et al.,^3^ for example, showed in a three-year randomized clinical trial that the additional application of fluorides (fluoride-salt, fluoride varnish, fluoride lozenges) did not result in a decreased caries incidence and prevalence.^3^ Additionally, there is limitation on the use of fluoride treatment in toddlers and children, because of the risk of fluorosis if swallowed.^4,5^ This underlines the need for novel strategies and active ingredients for preventive oral health care products which can be used either alone or in addition to fluorides and are safe if swallowed.

It was reported that dental plaque of caries-free children has higher levels of calcium and phosphorus compared to caries-active children.^6^ Thus, the use of synthetic calcium phosphates as active ingredients in oral care products seems to be a promising biomimetic approach to positively influence the remineralization-demineralization process towards net remineralization and consequently to prevent caries.^7–12^ One of these calcium phosphates that can be used in oral care products is synthetic hydroxyapatite (Ca_5_(PO_4_)_3_OH; HAP).^13^ HAP shows a high similarity to the mineral phase of human teeth and bones.^4,13,14^ The main key advantage of synthetic HAP is its high biocompatibility.^4,14^ Thus, HAP as active ingredient is well-suited for oral care formulations for all age groups, including infants and toddlers because of the absence of any fluorosis-risk.^4,5^ A recent in situ study showed that a HAP toothpaste remineralized initial enamel caries and inhibited enamel demineralization as effective as a toothpaste with 500 ppm fluoride provided as amine fluoride. In contrast to the amine fluoride toothpaste, the microradiographic images showed that the HAP toothpaste also remineralized deeper enamel layers.^11^ Besides in situ investigations, clinical trials have shown caries-preventing effects of HAP-based toothpastes.^12,15^ In addition to its remineralizing properties, HAP has been shown to reduce bacterial colonization on enamel surfaces.^16,17^ An in situ study showed that rinsing with a HAP-water-dispersion (i.e., a basic mouthwash without additives) reduced bacterial attachment to the tooth surface as effective as the (antibacterial) gold standard chlorhexidine. Unlike chlorhexidine, HAP does not kill oral bacteria, but shows anti-adhesive characteristics.^16^ Taken together, all these properties make HAP a promising biomimetic active ingredient for daily oral care applications.

In addition to daily toothbrushing, fluoride gels with high fluoride concentrations (e.g., 12,500 ppm F^−^) are frequently recommended by dentists for high caries-risk individuals.^18^ A Cochrane review reported caries reduction of 21% (95% CI: 14–28%) when using fluoride gels compared to placebo gels.^19^ However, using such high-concentrated fluoride products increases the risk for the development of dental fluorosis or other side effects, especially in children when different fluoride sources (e.g., fluoride gel, fluoride toothpaste, fluoride mouthwash, fluoride lozenges, fluoridated salt etc.) are combined.^20,21^ Consequently, highly concentrated fluoride gels should not be used on a daily basis as daily oral care routine at home. However, daily oral health care regimen has been shown to have positive effects on oral health.^22^ Furthermore, fluoride oral care gels seem to be more effective when acidulated,^23^ thus posing the risk of erosive tooth wear if used frequently. In contrast, HAP particles interact with tooth surfaces even under neutral conditions,^13,16,24^ and HAP-based toothpaste and gel has been shown to release calcium and phosphate ions in dental plaque,^25,26^ hence the potential to promote remineralization and inhibit demineralization of tooth tissue. Thus, it is conceivable that the daily usage of a fluoride-free, hydroxyapatite-based oral care gel is a promising strategy to support the caries preventive effect of toothpaste.

The aims of the present in vitro study were to investigate if a simulated daily use of a HAP-based oral care gel containing 15% HAP remineralizes early caries lesions (1) more effectively than artificial saliva alone, and (2) as effective as the weekly use of a high fluoride concentration oral care gel containing 12,500 ppm fluoride. Our hypotheses are that each of the two gels promotes remineralization that is significantly greater than zero, and that neither gel is inferior to the other with respect to promoting the remineralization.

## Materials and methods

### Specimen preparation

Sound bovine teeth were collected and sterilized in accordance with the university procedure for sterilization of teeth used for studies. Following sterilization, the teeth were cleansed of soft tissue debris, brushed with pumice slurry and an electric toothbrush, and then examined by transillumination. Teeth without cracks, hypoplasia, white spot lesions and other malformations were selected and stored in distilled deionized water saturated with 0.1% thymol during the sample preparation process. Using a water-cooled cutter, tooth blocks (approximately 3 mm length × 3 mm width × 1.5 mm thick) were produced from the buccal surface of each bovine tooth, and a total of 60 blocks were produced. Following this, all surfaces of each block were painted with two coats of acid-resistant nail varnish, except the enamel surface. The prepared specimens were stored at 100% relative humidity at 4 °C until use. All specimens were prepared by the same trained technicians using standard operating procedures.

### Caries lesion formation

Early caries-like lesions were created on the exposed enamel surface on each tooth block by subjecting the blocks to 4 days demineralization in an acidified gel system.^27^ The gel was prepared by adding 0.10 M sodium hydroxide to 0.10 M lactic acid to give a final pH value of 4.5. To this solution, 6% ^w^/_v_ hydroxyethyl cellulose was added whilst vigorously stirring. The final consistency of gel was a viscosity in the region of 100 cP.^27^ Demineralization periods were chosen based on prior experience and to create lesions with comparable data.^27^ Following lesion formation, the nail varnish was carefully and totally removed with acetone.

### Lesion baseline measurement

The baseline mineral loss (∆z) in the produced lesions were measured using transverse microradiography (TMR) as follows. A tooth slice (~150 µm thick) was cut from each tooth block using a water-cooled diamond wire saw. This slice served as baseline for determining the Pre-treatment TMR parameter (mineral loss (∆z_1_) of the lesion before remineralization (pre-test parameters). Also the baseline parameter was used to select the lesions that are suitable for the remineralization study. The pre-test slices were processed for TMR assessment as follows. First, both sides of the slice were polished using Adhesive Back lapping film in a MultiPrep™ Precision Polishing machine (Allied High Tech, USA) to achieve planoparallel surfaces as well as reduce the thickness of the slice to 100 µm (the appropriate thickness for TMR). Following this, the slices were microradiographed on a type lA high-resolution glass X-ray plate (Microchrome Technology, CA, USA) using a Phillips X-ray generator system set up for this purpose. The plates were exposed for 10 min at an anode voltage of 20 kV and a tube current of 10 mA, and then processed. Processing consisted of a 5 min development in Kodak HR developer and 15 min fixation in Kodak Rapid-fixer before a final 30 min wash period. After drying, the microradiographs were subjected to visualization and image analysis using a computer program (TMR2006 version 3.0.0.6). The hardware were a Leica DMR optical microscope linked via a Sony model XC-75CE CCTV camera to a personal computer. The enhanced image of the microradiograph was analyzed under standard conditions of light intensity and magnification and processed, along with data from the image of the step wedge, by the TMR program. By this method, the parameter of integrated mineral loss (vol%, µm) was quantified for each caries lesion.

### Preparation of treatment solutions

Standard remineralization and demineralization solutions were prepared using previously published protocols.^28–30^ The remineralization solution (artificial saliva) was composed of 3.8 ppm Mg^2+^ (MgCl_2_·6 H_2_O), 84.36 ppm PO_4_^3−^ (K_2_HPO_4_/KH_2_PO_4_) 50 ppm Ca^2+^ (calcium lactate), 0.05 ppm fluoride, 0.625 g/L KCl, 0.4 g/L carboxymethylcellulose, and 2 g/L methyl-4-hydroxybenzoate, with the pH adjusted to 7.2 using 1 M KOH. The demineralizing solution (DS) served as an acid challenge simulating the acids generated in the dental plaque with intake of cariogenic meal. The DS was composed of 2.0 mMol/L Ca(NO_3_)_2_·4H_2_O, 2.0 mMol/L KH_2_PO_4_, 75.0 mMol/L CH_3_COOH with the pH adjusted to 4.5 using 1 M KOH.

### Test products and study groups

The 60 tooth blocks were randomly assigned to the following three experimental groups (20 blocks/group); (A) hydroxyapatite oral care gel (Karex gelée, Dr. Kurt Wolff GmbH & Co. KG, Bielefeld, Germany) containing 15% HAP microclusters (crystallite size: length ≈ 80 nm (median) × width ≈ 30 nm (median)), (B) fluoride oral care gel (Elmex gelée, CP GABA GmbH, Hamburg, Germany) containing 12,500 ppm fluoride provided as amine fluoride, and (C) artificial saliva (untreated). The pH of each treatment product was measured and recorded. All study products were coded (“Gel A” and “Gel B”) by the sponsoring company, who retained the code until the completion of the study and data interpretation, thus blinding both the investigators and the subjects to the product identity. Using nail varnish, the 20 blocks in each group were attached on an acrylic cylindrical rod that was attached to the cover of a 250-ml treatment tube. The three groups were subjected to remineralization using a pH-cycling (demineralization/remineralization) model, simulating the activities within the oral environment as close as possible, to test the ability of each test group to remineralize the early enamel caries lesions.

### pH cycling Regimen^28^

24 h prior to commencement of treatment the samples were stored in saliva with constant gentle rotation to allow an artificial pellicle-like layer to form after which the treatment starts the next day. The cyclic treatment regimen consisted of once daily 2 h acid challenge in DS, while the remineralization treatment was in accordance with each product manufacturer’s instruction. The HAP gel was applied once every day, while the fluoride gel was applied once a week. For the rest of the 24 h in a day the samples were treated in fresh artificial saliva (AS). For application, a thin ribbon of gel was applied with a finger over the enamel surface of each tooth block and left for 3 min, after which the gel was removed gently with gauze, followed by rinsing under running deionized distilled water (DDW) for 30 s, and the samples placed back into AS. The samples in group C remained in AS throughout the study period. Fresh AS and DS were used each day (changed after the acid challenge period). For treatment in AS (remineralization solution) and demineralization solutions, 200 mL of the treatment medium (AS or DS) was placed into each 250 mL treatment tube. Artificial saliva treatments were magnetically stirred at 350 rpm, while the DS treatment was static. The gel and DS treatment phases were performed inside the incubator at 37 °C while the rest of the experimental phase was conducted at room temperature. The experiment ran for 28 days. On termination of the experiment, the blocks were harvested and processed for remineralization assessment using TMR.

### Post-treatment processing and study outcomes

Following pH-cycling, a tooth slice (~150 µm thick) was cut from each tooth block. The slices were processed for TMR as described above for the pre-test slices used for the baseline data. Although the pre-test slices have been microradiographed and analyzed for selection of the appropriate lesions, they were microradiographed again together with the post-test slices. This step enabled the pre-test and post-test slices to be microradiographed together and images developed and analyzed under the same conditions for generation of the pre-test (∆z_1_) and post-test (∆z_2_) mineral loss of the lesions as described for baseline slices above. In addition to the ∆z_1_ and ∆z_2_, this process also yielded the pre-test and post-test microradiographic images of the lesions. Using the microradiograms, the pattern and the extent of remineralization produced within each lesion by each treatment group was examined and described by comparing the pre-test and post-test images side-by-side. For each tooth block the post-test mineral loss was subtracted from the pre-test mineral loss, and then standardized across the group by dividing that difference by the pre-test mineral loss to obtain the % mineral gain (% remineralization). The three treatment groups were compared using the % remineralization.

### Power analysis and sample size calculation

Based on power analysis, the sample size calculations were performed using nQuery Advisor software (Statistical Solutions, Cork, Ireland). In previous remineralization studies that used similar pH-cycling model, the mean % remineralization was equal to 28.76 with a standard deviation equal to 7.2,^28,31^ and for the hypothesis that each of the two gels promotes remineralization that is significantly greater than zero, an effective sample size of 20 blocks will have power greater than 0.85 with a 0.05 one-sided significance level to detect a difference between a hypothesis mean of zero and a sample mean % remineralization equal to or greater than 10% using a two-sided t-test of two independent means.

## Statistical analysis and results

STATA software version 10.0 (StataCorp LP, USA) at a significance level of 5% was used. The pre-test mineral loss, post-test mineral loss, and difference (mineral gain) between the pre-post test scores were normally distributed (*p* > 0.05) across the three conditions (HAP gel, fluoride gel, and artificial saliva) as assessed by Shapiro-Wilk’s test. No outliers were found in the data, as assessed by inspection of a boxplot. However, the assumption of homogeneity of variances was violated (*p* < 0.001) as assessed by Levene’s test.

To determine any significant change (remineralization) made by the test product (intra-group comparison), three paired-sample t-tests were conducted to examine the change in mean values of the ΔZ_1_ and ΔZ_2_ across each of the three conditions. With the use of any of the test products, there was a statistically significant (*p* < .001) change (remineralization) in mineral loss between the pre-test and post-test (Table 1). To determine the efficacy and any significant difference among the conditions, a one-way Welch analysis of variance (ANOVA) test was conducted to examine the differences in percentage values, composed from the differences (mineral gain) between the pre-post test scores, among the three conditions (Table 2). Games-Howell’s multiple comparison test was then used to identify which conditions significantly differed from each other. The percentage value was statistically significantly different for different conditions, Welch’s *F*(2, 29.632) = 270.048, *p* < 0.001. There was a decrease in percentage value from HAP gel (39.266 ± 6.982) to artificial saliva (6.008 ± 2.623) with a mean decrease of 33.257, 95% C.I. [29.096, 37.419], which was statistically significant (*p* < 0.001). Similarly, there was a decrease in percentage value from fluoride gel (40.765 ± 10.745) to artificial saliva (6.008 ± 2.623) with a mean decrease of 34.757, 95% C.I. [28.528, 40.985], which was statistically significant (*p* < 0.001). Though there was a slight decrease in percentage value from fluoride gel (40.765 ± 10.745) to HAP gel (39.266 ± 6.982) with a mean decrease of 1.499, 95% C.I. [−5.536, 8.534], it was not statistically significant (*p* = 0.861).

**Table 1:** The pre-test and post-test mineral loss (vol%.µm) in each experimental group.

Test products	pH	Pre-test ∆z	Post-test ∆z	Change (95% CI)	*t(19)*	*P* value	*d*
HAP gel	7.8	1713 ± 130.23^a^	1016.50 ± 153.39^b^	696.50 (640.189–752.811)	25.888	<0.001*	5.789
Fluoride gel	6.8	1726 ± 104.95^a^	1030 ± 232.18^b^	696 (622.187–769.647)	19.78	<0.001*	4.423
Artificial saliva	7.2	1759 ± 130.80^a^	1652.25 ± 122.89^d^	106.25 (83.563–128.936)	9.803	<0.001*	2.192

*d* Cohen’s d, an effect size.

*Difference statistically significant.

^a^No statistically significant difference between the groups.

^d^Significantly difference from HAP and fluoride gels.

**Table 2:** Percentage remineralization (mineral gain) in each of the treatment groups.

Test products	Mean	Std. deviation	95% confidence interval for mean		Minimum	Maximum
			Lower bound	Upper bound		
HAP gel	39.2657	6.98194	35.9980	42.5333	25.97	55.83
Fluoride gel	40.7648	10.74508	35.7359	45.7936	22.78	59.21
Artificial saliva	6.0082	2.62307	4.7806	7.2359	1.17	11.23
Total	28.6796	17.79961	24.0814	33.2777	1.17	59.21

Finally, the primary endpoint was to check for non-inferiority of the HAP gel to the fluoride gel. Non-inferiority/equivalence was established if the difference between the two gels in their percentage remineralization was not regarded to be clinically relevant and was set to Δ ≤ 20%. In the non-inferiority analysis, the significance testing was used to parallel the non-inferiority margin.^32^ Establishing superiority between HAP gel versus artificial saliva and fluoride gel versus artificial saliva was conducted first. Confidence intervals were generated at 95% and 80% to examine if the lower and upper bounds were above zero. The difference between HAP gel and artificial saliva had a 95% confidence interval of 29.096 to 37.419 and an 80% confidence interval of 30.302–36.213. The difference between fluoride gel and artificial saliva had a 95% confidence interval of 28.528–40.985 and an 80% confidence interval of 30.352–39.161. All four confidence intervals established the superiority of HAP gel over artificial saliva and fluoride gel over artificial saliva at both the 5% level of significance and 20% level of significance, respectively. Confidence intervals were then generated at 95% and 80% to examine the difference between HAP gel and fluoride gel. Results yielded a 95% confidence interval of −8.533–5.535 and an 80% confidence interval of −6.533–3.535. Both confidence intervals established non-inferiority on the basis of a non-significant test of the null hypothesis (*p* = 0.861).

**Fig. 1:**
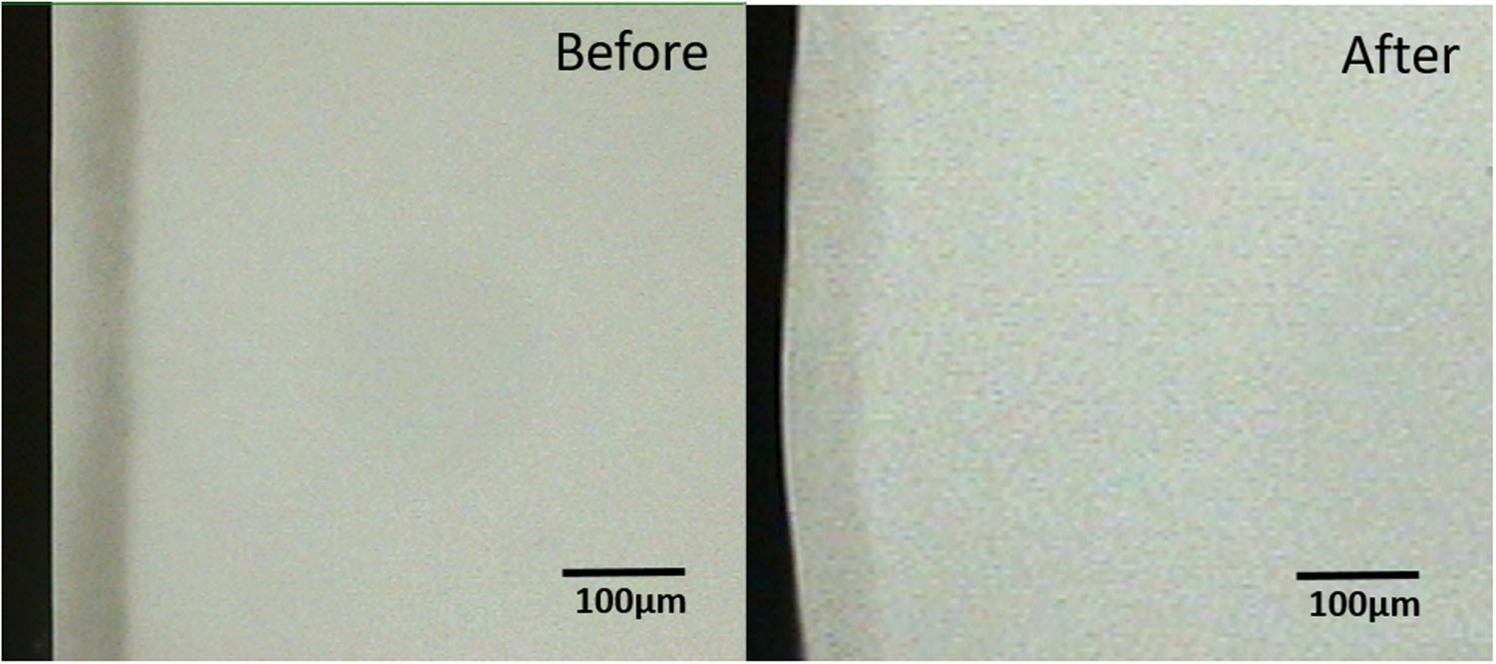
Remineralization by 15% HAP oral Care Gel. Representative microradiographic images of enamel subsurface lesions (Initial caries lesions), before and after remineralization with 15% HAP oral care gel.

**Fig. 2:**
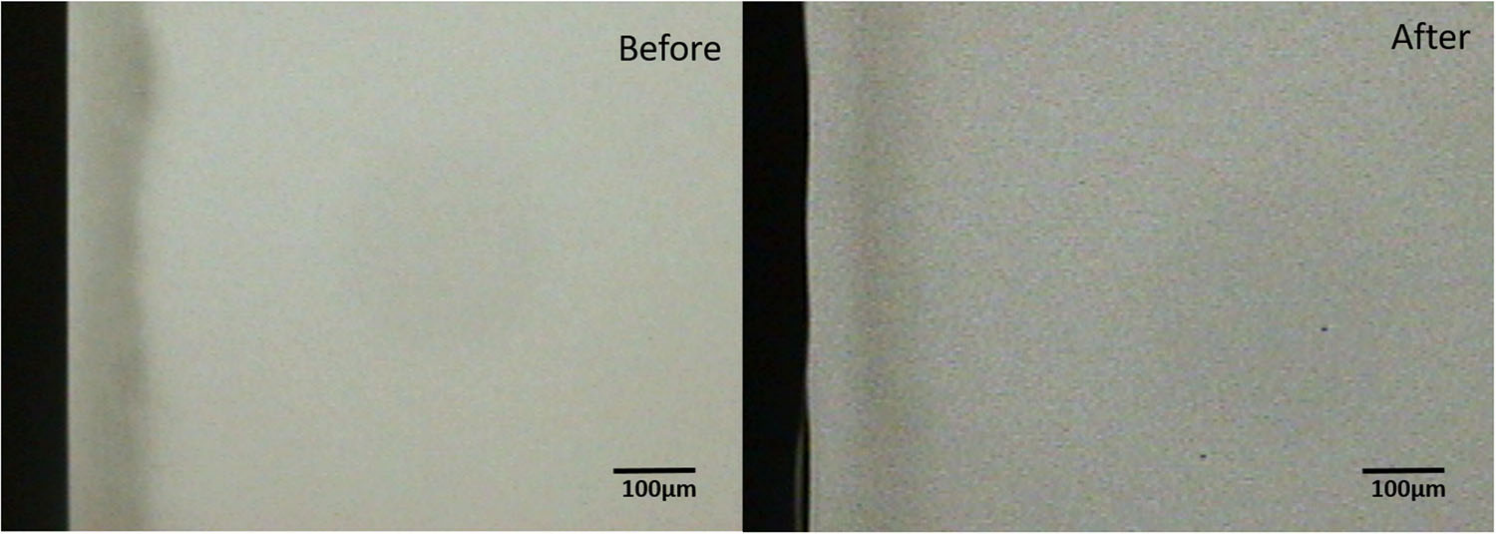
Remineralization by 12,500 ppm fluoride oral care gel. Representative microradiographic images of enamel subsurface lesions (Initial caries lesions), before and after remineralization with fluoride oral care gel containing 12,500 ppm fluoride.

**Fig. 3:**
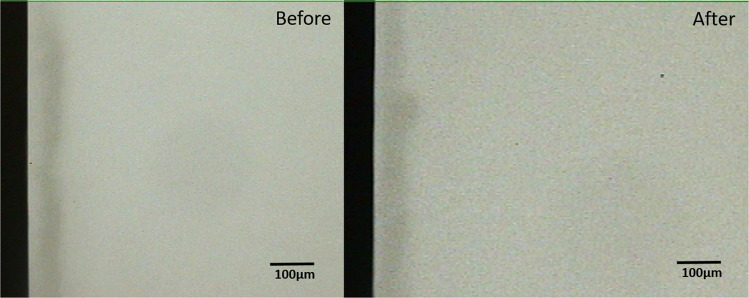
Remineralization by Artificial Saliva. Representative microradiographic images of enamel subsurface lesions (Initial caries lesions), before and after remineralization with artificial saliva.

Comparison of the pre-test and post-test microradiograms from each experimental group confirmed the remineralization indicated by the statistical data (Figs. 1–3). Critical examination of the images shows that the remineralization induced by the fluoride gel was limited to the outer half (surface zone) of the lesion without reduction of the depth of the lesion (Fig. 2), while HAP induced a more homogenous remineralization distributed throughout the entire thickness of the subsurface lesion with a resultant reduction in the depth of the lesion (Fig. 1). Remineralization induced by the artificial saliva was not clearly obvious from the images (Fig. 3). These remineralization patterns were consistent in all specimens in their respective experimental group.

## Discussion

Having been established through clinical as well as in situ and in vitro studies that hydroxyapatite microclusters or nanoparticles in vehicles such as toothpaste and mouthrinse are as effective as fluoride in promoting caries remineralization and inhibiting tooth tissue demineralization,^8–12,15,16^ the present study investigated if daily use of a hydroxyapatite-based oral care gel containing 15% HAP remineralizes early caries lesions as effective as weekly use of an established and commercially-available high fluoride concentration oral care gel containing 12,500 ppm fluoride. The use of the gels was simulated using a net-remineralization pH-cycling caries model. A pH-cycling caries model serves as a bridge to in vivo caries studies as they mirror clinical conditions, where demineralization and remineralization alternate constantly (i.e., pH cycling) and are only interrupted during the very short period of application of investigational products.^29,30^ pH cycling models have long been developed and described in the literature over the years, and have been accepted and utilized in dental sciences and the industry as an appropriate alternative to animal testing, particularly for ionic fluoride-based dentifrices.^33–35^ One may argue that this model lack the cariogenic microbial biofilm that is a key factor in the caries process; however, the alternating feast and famine episodes that occurs in biofilm in the oral cavity is mimicked by alternating exposure of the samples to demineralization and remineralization solutions.^28^

With 39% and 41% remineralization by HAP and fluoride gel respectively, the result of the present study accepted our hypotheses that each of the two gels promotes remineralization that is significantly greater than zero, and that neither gel is inferior to the other with respect to promoting the remineralization. This result is in agreement with the reports of two recent clinical non-inferiority studies on HAP containing toothpaste. ^36,37^ One is a randomized controlled clinical trial that investigated enamel caries progression in highly caries-susceptible orthodontic patients, and demonstrated the non-inferiority of toothpaste containing 10% microcrystalline HAP to that containing 1400 ppm fluoride provided as amine fluoride and stannous fluoride.^12^ The other is an in situ study that demonstrated the non-inferiority to each other, of two children toothpaste formulations based 10% HAP and 500 ppm fluoride respectively, for promotion of caries remineralization and inhibition of tooth demineralization.^36^ Also in agreement with the present study is the result of a similar net remineralization pH cycling study that reported 10.56% and 9.58% remineralization, as measured by lesion depth reduction, by 10% HAP and 1450 ppm amine fluoride toothpastes respectively.^37^ In another similar study that measured remineralization by percentage surface microhardness recovery of initial caries lesion, 46.27% and 28.84% remineralization were reported for 10% HAP toothpaste and 1.23% acidulated phosphate fluoride gel respectively.^38^ In these two studies, there were no statistically significant differences between the HAP and the fluoride oral care formulations.^37,38^ The results of these previous studies and the present study can be attributed to the established modes of action of HAP as anti-caries and caries remineralizing agent.^8^ It is well-established that HAP has a high biocompatibility with a strong affinity to tooth tissues, and as such adsorption to tooth surfaces.^39^ Particulate HAP when adsorbed on tooth surface induce remineralization of initial caries lesions by directly filling micropores in demineralized tooth tissue,^40,41^ where it acts as a crystal nucleus, and promotes crystal deposition and growth by continuously attracting large amounts of calcium and phosphate ions from the surrounding remineralization solution.^41,42^ A recent study demonstrated that the HAP oral care gel tested in the present study released calcium and phosphorus on application on tooth surfaces,^26,43^ thus further highlighting the potential of this gel formulation to influence remineralization and demineralization process on tooth surface. The effectiveness of fluoride in remineralizing early caries lesions in the present study once again contributed to the wealth of evidence supporting fluoride as having the most consistent benefit in preventing caries and remineralizing initial caries lesions.^44–47^

It is pertinent to mention that while previous studies compared 10% HAP with standard fluoride concentration (500–1450 ppm),^12,35^ the present study compared 15% HAP with high fluoride concentration (12,500 ppm) formulation (prescription strength) that are recommended for individuals at high risk of dental caries,^48,49^ thus 15% HAP achieved comparable efficacy with a prescription-strength high fluoride concentration in remineralizing initial caries. This suggest that 15% HAP oral care gel, when used daily, can serve as an alternative to high fluoride concentration products prescribed for weekly application. Caries preventive agents should preferably be used on a daily basis to maintain a preventive concentration of the agent in oral cavity at all times.^47^ Furthermore, on a risk/benefit ratio, while high fluoride concentration dentifrices can only be used by adults due to the risk of fluorosis in children, ^20,50^ HAP-based dentifrices and gels at any concentration can be used by all ages since no adverse effect has been reported in previous clinical studies.^4,12,35^ This would go further to eliminate having different dosages for infants, children and adults, as it is the case with fluoride at the moment.^48,49^

Although significantly lower than those observed with the gels, the artificial saliva used in the present study was able to induce a statistically significant level of remineralization of initial caries lesion. This confirmed the artificial saliva used in the present study to mimic natural saliva that has, but limited, capacity to induce remineralization of initial caries lesions and prevent caries development. ^51,52^ The present artificial saliva, with its neutral pH (7.2), behaved more like the natural saliva of low caries risk individuals that maintain higher resting pH value (hence higher degree of supersaturation with respect to apatite) that increases the remineralization potential of saliva in these individual than their high caries risk counterparts.^53,54^

The remineralization of the outer half of the lesions by the fluoride gel as shown in Fig. 2 has long been established in previous studies^55^ and confirmed in more recent studies.^12,56^. This effect is not peculiar to products with high fluoride concentration as used in the present study; it was recently reported in an in situ study that investigated children’s toothpaste with 500 ppm fluoride.^12^ It was also reported in a previous study that higher fluoride concentrations did not produce any further significant increase in remineralization, rather surface zone remineralization were apparent in lesions subjected to the 250- and 500-ppm F^−^ solutions.^55^ Also an investigation of the effectiveness of varying doses (500, 1000, and 1500 ppm) of fluoride in remineralizing initial caries in primary teeth further confirmed this surface zone lamination.^46^ It is conceivable that the lamination effect would place a limit to the dose-dependent effect of fluoride effectiveness, and as such at a certain concentration the effect plateaus and further increase may not improve the effectiveness.^57^ Besides, this has the negative effect of preventing complete and fuller remineralization of the deeper zone of the lesions over time. In contrast, the more homogenous remineralization, distributed throughout the entire thickness of the subsurface lesion, induced by the HAP gel (Fig. 1) may indicate that increasing the dose of HAP or continued usage of the toothpaste may result to increased remineralization of the lesion, and ultimately lead to complete or fuller remineralization of the initial lesion.

It is worth mentioning that a main limitation of the study is the fact that the pH cycling models, though mirror clinical conditions, lacks the biological processes within the oral cavity, particularly within the dental plaque milieu, which influences the remineralization potential of the oral care products. Furthermore, demineralization process used in pH cycling is more aggressive at each occasion than the acid attacks the tooth is exposed in the oral cavity. Therefore, future research should focus on clinical trials to further establish the effectiveness of HAP-based oral care gel for caries remineralization and prevention.

## Conclusions

- The present study confirmed that a fluoride-free oral care gel with 15% hydroxyapatite is as effective in remineralizing initial caries lesions as a highly concentrated fluoride oral care gel with 12,500 ppm F^−^, and neither gel is inferior to the other with respect to remineralizing initial caries lesions.
- Both gels were significantly more effective in remineralizing initial caries lesions than artificial saliva alone.
